# Iron, glucose and fat metabolism and obesity: an intertwined relationship

**DOI:** 10.1038/s41366-023-01299-0

**Published:** 2023-04-07

**Authors:** Catriona Hilton, Rugivan Sabaratnam, Hal Drakesmith, Fredrik Karpe

**Affiliations:** 1 Oxford Centre for Diabetes, Endocrinology and Metabolism, University of Oxford, Oxford, UK; 2 Steno Diabetes Center Odense, Odense University Hospital, Odense, Denmark; 3 Department of Clinical Research, University of Southern Denmark, Odense, Denmark; 4 MRC Human Immunology Unit, MRC Weatherall Institute of Molecular Medicine, University of Oxford, John Radcliffe Hospital, Oxford, UK

**Keywords:** Iron, Obesity, Type 2 diabetes

## Abstract

A bidirectional relationship exists between adipose tissue metabolism and iron regulation. Total body fat, fat distribution and exercise influence iron status and components of the iron-regulatory pathway, including hepcidin and erythroferrone. Conversely, whole body and tissue iron stores associate with fat mass and distribution and glucose and lipid metabolism in adipose tissue, liver, and muscle. Manipulation of the iron-regulatory proteins erythroferrone and erythropoietin affects glucose and lipid metabolism. Several lines of evidence suggest that iron accumulation and metabolism may play a role in the development of metabolic diseases including obesity, type 2 diabetes, hyperlipidaemia and non-alcoholic fatty liver disease. In this review we summarise the current understanding of the relationship between iron homoeostasis and metabolic disease.

## The intertwined circuits of iron and metabolic homoeostasis

Given the enormous range of cellular proteins requiring iron for correct energy transformation it is perhaps not surprising that iron availability would affect nutrient energy transport and storage, such as for glucose and lipid homoeostasis [1]. In this review we will examine the evidence to suggest that the roots of iron homoeostasis and energy metabolism have evolved to be closely knitted together. It can be hypothesised that energy substrate metabolism and iron sensing are co-regulated to ensure that iron is available for efficient energy use and, in times of excess, adequate storage of energy. We will see how components of the iron and erythropoietic pathways are also expressed in AT [2] and the endocrine pancreas [3] and how they modulate body fat accumulation [4, 5] and glucose and lipid homoeostasis [6], and also how obesity, glucose and lipids can modulate iron homoeostasis [7, 8]. This co-regulation may provide a safety net to provide iron for tissues during periods of starvation, but in the context of metabolic derangement this relationship can break down and be counterproductive. Equally, diseases characterised by iron-loading lead to metabolic disease [9].

## Regulation of iron absorption and transport

In humans, iron is essential for erythropoiesis, for formation of myoglobin for oxygen transfer to muscle, host defence, DNA replication and repair, for numerous metabolic enzymes and for mitochondrial energy production. Conversely, excess iron is toxic: ferrous iron forms highly reactive hydroxyl free radicals via the Fenton reaction that cause damage to cellular components including lipids, DNA and proteins, ultimately leading to tissue damage. Appropriate iron balance is crucial for optimum cell function. Both iron depletion [10] and iron overload [11] negatively affect mitochondrial oxidative phosphorylation.

**Fig. 1:**
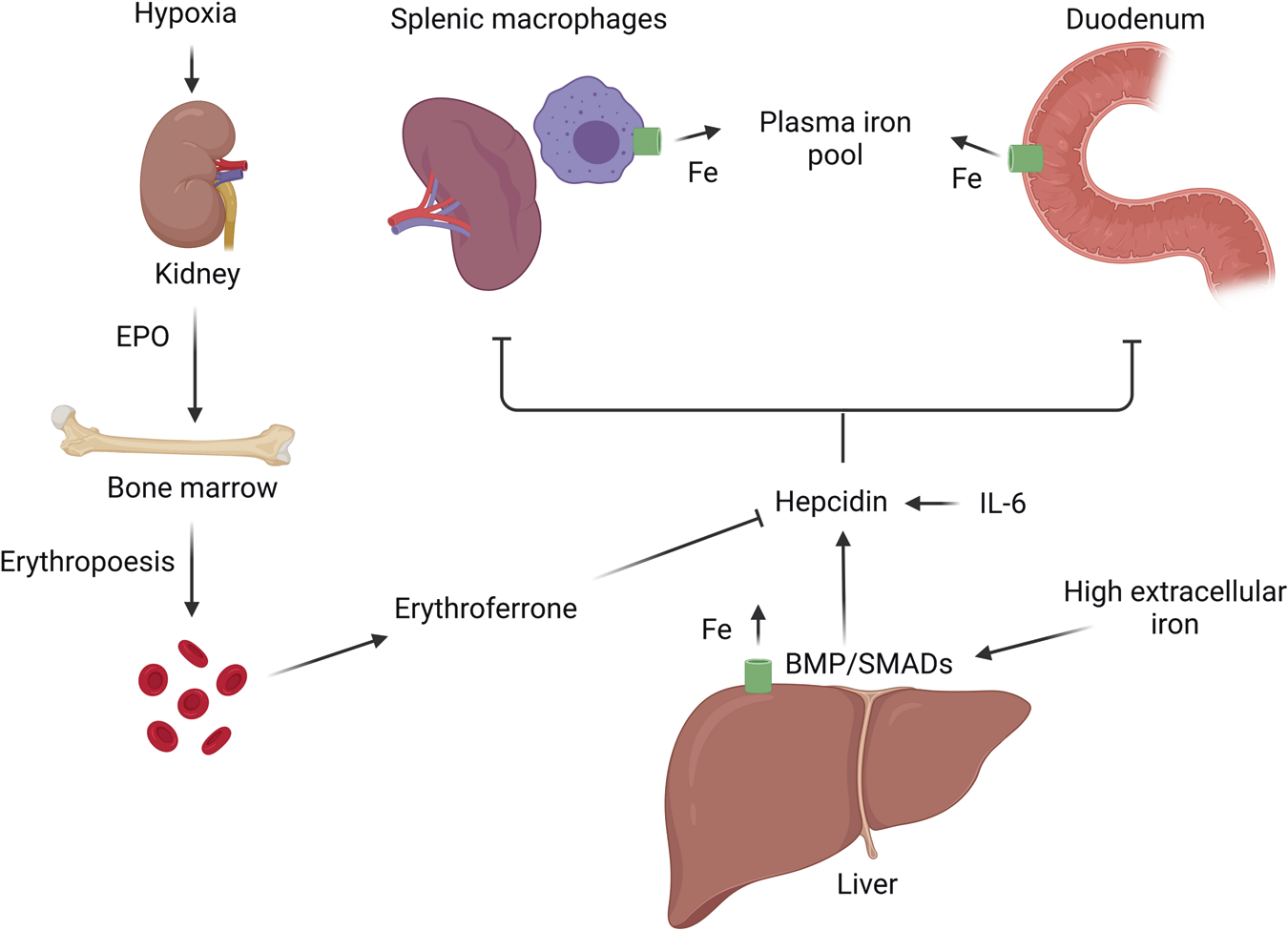
A summary of human iron regulation. Hepcidin, produced primarily by hepatocytes, reduces iron efflux from cells and duodenal iron absorption by reducing the stability of the ferroportin transporter (green). Hepcidin is mainly regulated via the bone morphogenic protein (BMP)/SMAD signalling pathway. The inflammatory cytokine interleukin-6 (IL-6) induces hepcidin. In response to cellular hypoxia the kidneys release erythropoietin (EPO), which stimulates erythropoiesis and the production of erythroferrone from erythroblasts. Erythroferrone inhibits hepcidin release, thereby increasing iron absorption and release of iron from stores. Image created in Biorender.com.

The mechanisms regulating iron absorption, storage and release are summarised in Fig. 1. Full discussion of the complex pathways controlling iron homoeostasis is beyond the scope of this review and is outlined in detail elsewhere [12]. Because humans do not have a regulated mechanism to excrete iron, uptake from dietary sources must be carefully regulated to balance losses. Macrophages and hepatocytes have an enhanced ability to store iron and act as a buffer of iron stores. Iron can be liberated from stores and exported from cells via the ferroportin transporter.

Hepcidin is an iron-regulating peptide hormone that is produced chiefly by hepatocytes and considered the master regulator of iron homoeostasis [12]. It is expressed to a lesser extent by other tissues including pancreatic β-cells [3] and adipose tissue (AT) [2]. It is released in response to rising iron saturation of plasma transferrin, increased hepatic iron stores, or inflammation, and negatively feeds back to reduce iron availability by causing degradation of the ferroportin transporter, thereby reducing iron uptake by enterocytes and inhibiting release of iron from macrophage and splenic stores. Hepcidin is mainly regulated via the BMP/SMAD signalling pathway [13]. Bone morphogenic proteins (BMPs) phosphorylate SMAD1/5/8 that, on binding to SMAD4, translocate to the nucleus to activate target genes, including hepcidin [13, 14]. The inflammatory cytokine interleukin (IL)-6 induces hepcidin [15] through the phosphorylation of STAT3 [15]. In response to infection or inflammation hepcidin rises, causing iron to be sequestered as a defence against siderophilic bacteria [16]. In response to acute haemorrhage or erythropoietic stress erythroferrone (ERFE) is produced and released by bone marrow erythroid progenitor cells. ERFE inhibits hepcidin release via actions on the BMP pathway [17], taking the brakes off iron absorption and releasing and supplying more iron for synthesis of haemoglobin [18].

## Iron status in obesity and fat distribution

Since the 1960’s it has been recognised that people with obesity have lower serum iron [19]. Children and adolescents with overweight and obesity are twice as likely to be iron deficient, with prevalence of iron deficiency increasing with body mass index (BMI) [20]. Pregnant women with obesity have impaired iron absorption in late pregnancy, and their offspring have reduced iron stores aged six months [21]. Adults with obesity are also at higher risk for iron deficiency [22, 23] which is common in patients referred for bariatric surgery [24]. This may be partly explained by dietary patterns. The ‘double burden’ of overweight and obesity with concurrent undernutrition and micronutrient deficiencies is well-recognised as a public health problem [25]. However, people with obesity have higher hepcidin levels which rise with BMI [22], implying that the lower serum iron parameters are not purely dilutional or nutritional.

Beyond absolute obesity, body fat distribution also associates with iron parameters. Cross-sectional studies have shown that transferrin saturation negatively correlates with BMI [23] and is lower in men and women with obesity and an expanded waist circumference than in those with obesity but with a smaller waist circumference [23]. Serum iron negatively correlated with waist circumference in Hispanic women (but not men) [26] and ferritin positively associated with waist-to-hip ratio (WHR) independent of BMI [27] and with visceral fat area [28] and with waist-to-thigh ratio in healthy men [4]. In another study, women with overweight or obesity and central adiposity had higher hepcidin, lower transferrin saturation and impaired absorption of supplemental (but not dietary) iron [29]. Gynoid fat mass does not have an association with iron metabolism [29]. A possible mechanism by which central obesity has this effect on iron status could be its association with systemic low-grade inflammation [30]. IL-6 upregulates hepcidin directly via hepatic STAT3 signalling in the liver [15, 31]. Interestingly, hepcidin expression in AT also increases with obesity [2]. The sex hormone oestrogen is strongly associated with body fat distribution and directly suppresses hepcidin [32], possibly to upregulate iron absorption to compensate for that lost through menstruation.

Obesity is an inflammatory state characterised by low-grade chronic activation of the non-specific immune system [30, 33]. Overnutrition and obesity cause a switch in the resident immune cells to an inflammatory phenotype with increased secretion of pro-inflammatory cytokines such as tumour necrosis factor alpha (TNFα), IL-1, and IL-6 [30]. The low-grade systemic inflammation associated with obesity contributes to the development of obesity-related metabolic disease [34].

Bariatric surgery is a model for rapid weight loss and several studies have examined the temporal effect on iron regulation following surgery. In a cohort of 20 postmenopausal women, bariatric surgery led to an average reduction of BMI from 48 kg/m^2^ to 40 kg/m^2^ accompanied by a fall in c-reactive protein (CRP) from 11 mg/l to 6 mg/l at 6 months. Serum hepcidin fell to less than a third of original levels [22]. This was associated with a fall in serum transferrin receptor levels and a rise in haemoglobin concentration. Weight loss induced by bariatric surgery leads to improved iron absorption in iron deplete individuals, but does not seem to alter iron absorption in those with adequate iron stores [35]. Within AT, bariatric surgery-induced weight loss led to increased expression of transferrin and decreased expression of the transferrin receptor, ferritin, and ferroportin [36].

Animal models allow us to examine the temporal relationship between iron and the development of insulin resistance in a more controlled manner. In mice fed a high fat diet, the development of insulin resistance preceded the development of disrupted iron metabolism in liver and visceral AT (VAT) [8].

### Iron status in adipose tissue development and function

In mice, an iron-rich diet led to smaller adipocyte size and reduced mass of epididymal AT (the murine equivalent of VAT), with no change in subcutaneous AT mass [5], with subsequent effects on insulin resistance and triglycerides. However, conversely, restriction specifically in epididymal fat was seen in mice treated with an iron chelator [37], accompanied by reduced AT inflammation and macrophage infiltration.

The metabolic effect of dietary iron-loading might depend on whether concurrent obesity is present. In ob/ob obese mice, a low iron diet or iron chelation therapy protected against β-cell failure and insulin resistance and lowered triglycerides, but these effects were not replicated in healthy weight mice [38]. This phenotype was the opposite of that seen in human haemochromatosis, an iron-loading disease (discussed below).

Iron load has local effects in AT. In acute iron overload, AT seems to be more sensitive to iron accumulation than liver [39]. In a mouse model where adipocytes were selectively rendered iron deficient by adipocyte-specific deletion of transferrin receptor-1, inguinal and brown AT reduced in mass via apoptosis [40]. Mice were subsequently protected from high fat diet induced obesity and metabolic deterioration, as indicated by improved insulin sensitivity and protection from hepatic steatosis. Adipocytes differentiate from mesenchymal stem cell precursors. Iron overload affects balance of differentiation of osteoblasts *vs* osteoclasts (derived from the same progenitors as preadipocytes), although it is not clear yet how this relates to adipogenesis [41]. In an in vitro study using rat adipocytes, incubation with transferrin or iron increased lipolysis and decreased insulin stimulated glucose uptake [42].

### Iron and adipokines

Adipokine release is influenced by iron homoeostasis. Acute iron overload in vitro and in vivo in mice leads to decreased expression of adiponectin and leptin [39, 43, 44]. Conversely, phlebotomy of humans with hyperferritinaemia and impaired glucose tolerance caused an increase in adiponectin [43]. Adiponectin is known to associate with lipid profile [45], although it remains unclear whether this is a causal relationship. Acute iron overload led to increased serum triglycerides and low‐density lipoprotein (LDL) cholesterol levels and reduced high‐density lipoprotein (HDL) cholesterol, which was partially mitigated by treatment with adiponectin and leptin, suggesting other mechanisms are also relevant.

Equally, as well as iron influencing adipokine release, leptin also stimulates hepatic hepcidin release through the JAK2/STAT3 signalling pathway [46].

### Iron status and adipose tissue macrophage function

Within murine AT, Prussian blue staining has revealed iron stores to be concentrated within macrophages, with a subpopulation having increased iron content and enhanced expression of genes involved in iron-cycling [47, 48]. AT macrophages are important for determining AT homoeostasis and the metabolic consequences of obesity [30]. Splenic macrophages are known to be major contributors to systemic iron levels by phagocytosing and releasing iron [12]. Within AT, a subpopulation of M2 (anti-inflammatory) macrophages exist that are iron-rich and can take up excess iron and release it in response to local deficiency [47]. These protect AT from iron overload [48]. There is a hypothesis with accumulating evidence that these can help control iron levels in the microenvironment. In obesity there is a shift in macrophage polarity towards the more inflammatory M1 phenotype [30]. There are also fewer iron-cycling M2 macrophages, and those present have impaired iron-handling abilities [47]. The impaired iron-handling ability of AT macrophages in obesity leads to accumulation of AT iron and deficiency of hepatic iron, along with elevated ferritin and transferrin [47]. Iron-handling genes in macrophages are also impaired by saturated fatty acid treatment in vitro [47].

### Iron and adipose tissue mitochondrial function

Finally, iron is vital for mitochondrial function. Mitochondria are intracellular organelles which are essential for energy metabolism, but also have diverse roles in cellular apoptosis, autophagy, cell growth and immunity [49, 50]. Different tissues have different densities of mitochondria; white AT has few mitochondria, reflecting its role as a reservoir for energy storage. Both iron deficiency and iron excess can cause mitochondrial damage and dysfunction [51]. Mitochondrial dysfunction in white AT has been implicated in the biogenesis of metabolic disease [52].

MitoNEET is a mitochondrial protein located in the outer mitochondrial membrane. It regulates iron transfer into the mitochondria, acting as a rate limiting step for the electron transport chain [53]. Mice with adipose tissue-specific overexpression of mitoNEET exhibited massive expansion of adipose tissue, but with preserved insulin sensitivity [53].

In contrast to white adipocytes, brown and beige adipocytes are important for non-shivering thermogenesis [54] and in line with this have high mitochondrial density [55]. Brown AT depots have been identified in humans as well as animals [56]. The iron requirements of brown AT are higher than for white AT and increase when brown AT is stimulated, and iron availability is crucial for the differentiation of brown/beige adipocytes [55].

## Effects of iron status on metabolic rate and satiety

Iron intake and stores might influence body weight by altering basal metabolic rate; iron deficient people and animals have impaired glucose turnover and impaired thermoregulation [57]. The hypothalamus plays a crucial role in appetite and regulation of food intake. Interestingly, people with obesity have more iron deposition in the hypothalamus than healthy weight controls, and this correlates with hepatic iron [58]. Iron deficiency is associated with reduced appetite, whilst iron supplementation is associated with increased appetite in children [59].

Iron overload or depletion also regulates leptin, with subsequent effect in appetite and food intake [44]. Iron deficiency is associated with reduced thyroid function [60]. Iron deficiency anaemia is also associated with reduced release of insulin-like growth factor-1 (IGF-1) [61], although it is less clear if this is due to anaemia or to iron deficiency per se.

## Iron-regulatory pathways and adipose tissue function

### Erythroferrone

Studies in mice have shown that Erfe is released from muscle in response to exercise and feeding [62]. In humans, ERFE increases in response to exercise training [63] and acute exercise [64] and following weight loss due to laparoscopic sleeve gastrectomy [65]. Conflicting associations have been reported with obesity and WHR [65, 66]. Work from our group has shown that BMP2 is a differentiation factor for subcutaneous abdominal, but not gluteal, adipocytes. In in vitro experiments ERFE inhibited BMP2 signalling in subcutaneous abdominal preadipocytes [67]. In mice, recombinant Erfe lowers circulating free fatty acids by increasing uptake into adipocytes and hepatocytes [63].

### Erythropoietin

Erythropoietin (EPO) is a hormone secreted by the kidneys in response to relative hypoxia. It acts on the bone marrow to stimulate production of red cells and drives production of ERFE, and therefore iron absorption. In mouse models, treatment with EPO or constitutive overexpression of EPO led to reduced blood glucose levels, lower glycated haemoglobin (HbA1c) [68] and reduced triglycerides by enhancing lipid catabolism within AT [69]. EPO treatment of humans led to improvements in lipid profile [69] and improved glucose tolerance [70, 71], with a rebound in fasting glucose when EPO was discontinued [71].

EPO is known primarily as a stimulator of erythropoiesis, but interestingly the EPO receptor is found in many other tissues and is highly expressed in white AT (WAT) and in the hypothalamus [72]. Knock out of the EPO receptor in non-hematopoietic tissues in animal models results in increased fat mass without affecting lean mass [72] and treatment of mice with EPO, or forced EPO overexpression, protects against obesity and improves glucose tolerance [68, 70]. Hypothalamic expression of EPO suggests that it may regulate energy balance centrally, but this has not been explored.

Some experimental evidence points towards a local role in AT. In vitro, treatment of the 3T3-L1 pre-adipocyte cell line with EPO suppressed adipogenesis [73]. EPO treatment is associated with reduced AT inflammation [73], and EPO reduces activation of cultured macrophages [73]. The fact that EPO has such marked metabolic effects is fascinating and suggests an evolutionary advantage to regulating energy homoeostasis in times of haemopoietic stress or according to iron availability.

## Iron overload syndromes and metabolic dysfunction

One of the first descriptions of insulin resistance was in 1929 in the context of ‘bronze diabetes’ [74], a disorder of iron loading now known as haemochromatosis. Since then, it has repeatedly been observed that diseases characterised by disordered iron metabolism tend to have a metabolic phenotype.

The thalassaemias are inherited haemolytic anaemias caused by deficient synthesis of either the α-chain (as in α-thalassaemia) or β-globulin chains (in β-thalassaemia) that make up the adult haemoglobin tetramer [75]. The unpaired globulin chains precipitate, leading to shortened red cell life span. Immature erythrocytes accumulate in the bone marrow and anaemia causes them to release ERFE, which inhibits hepcidin production, leading to increased iron absorption and ultimately iron overload. Both of the heterozygous α-thalassaemia [76] and β-thalassaemia [77] carrier states are associated with increased risk of insulin resistance and type 2 diabetes (T2DM). Indeed, 20–30% of β-thalassaemia major patients have diabetes, which is characterised by both insulin resistance and relative insulin deficiency due to beta cell failure [78]. Postulated mechanisms for this are discussed below.

Haemochromatosis is an inherited disease primarily caused by a mutation in the HFE gene [79], leading to constitutive activation of iron absorption. Haemochromatosis is characterised by iron overload in tissues, leading to liver cirrhosis. Haemochromatosis is associated with reduced BMI [9]. Paradoxically, people with haemochromatosis also have an increased risk of diabetes [9, 80], caused both by insulin resistance and decreased β-cell function [81], and increased rates of primary hypertriglyceridemia [82]. In one retrospective study, 31% of a cohort of patients with haemochromatosis had concurrent hypertriglyceridaemia, and therapeutic phlebotomy more than halved triglycerides in affected individuals [83], suggesting a direct effect of iron overload on the development of hypertriglyceridaemia. However, in another study of individuals without haemochromatosis but with hypertriglyceridaemia and hyperferritinaemia, therapeutic venesection to deplete iron stores had no effect above standard lipid lowering therapy [84].

Ferroportin disease is caused by mutations in ferroportin-1 gene. The classical phenotype is characterised by raised ferritin, normal transferrin and iron overload in macrophages, and in the non-classical phenotype ferritin and transferrin are both raised and iron overload affects the liver as well as macrophages [85]. Interestingly, ferroportin disease has not been reported to associate with an increased risk of diabetes or lipid disorders.

The influence of iron status on metabolic health is demonstrated in dysmetabolic iron overload syndrome (DIOS). DIOS refers to mild iron overload associated with features of the metabolic syndrome (T2DM, essential hypertension, non-alcoholic fatty liver disease (NAFLD) or polycystic ovary syndrome) in the absence of an identifiable cause for iron excess. Beyond the cardiovascular risk this confers, iron overload also independently predisposes to atherosclerosis by causing endothelial dysfunction [86].

Alcohol excess is commonly associated with iron overload and with abnormalities in iron metabolism [87] and individuals with alcoholic liver disease often have increased hepatic iron stores [88]. The increase in hepatic iron associated with alcohol excess leads to free radical mediated cellular damage and exacerbates the direct hepatotoxicity from alcohol. Both acute and chronic alcohol excess cause downregulation of hepcidin, leading to increased duodenal iron absorption. Hepcidin is downregulated both by direct action on the BMP/SMAD pathway [89] and hepcidin gene (HAMP) promotor binding [90].

## Iron stores, iron status and type 2 diabetes

Beyond the metabolic dysfunction seen in iron-loading diseases, there is an association between iron stores within the physiological range and risk of T2DM. Diabetes can be caused by a relative insulin deficiency, often in the face of insulin resistance. In an observational study of more than 6,000 men and women, higher fasting ferritin levels were associated with both impaired pancreatic β-cell function and decreased insulin sensitivity [91]. Similarly, diabetes associated with the thalassaemias [78] and haemochromatosis [81] is caused by both insulin deficiency and insulin resistance.

In a meta-analysis of eleven studies carried out in the USA and Europe containing 620 participants, individuals with T2DM had lower haemoglobin despite elevated iron stores, as indicated by elevated ferritin. Individuals with T2DM had elevated hepcidin, which was further elevated with concurrent obesity, and also had elevated hepcidin:ferritin ratio [92]. In agreement with this, one group recruited cohorts of individuals without diabetes, with prediabetes or with T2DM on insulin [93]. The group with T2DM on insulin had higher hepcidin than controls, whilst paradoxically the group with prediabetes had lower hepcidin levels than the control group. In another study recruiting matched cohorts, hepcidin and hepcidin: ferritin ratios were lower in people with T2DM and with polycystic ovary syndrome when compared with controls, but not in type 1 diabetes (T1DM), implying a role for iron overload in insulin resistance rather than insulin deficiency [94]. In a study of Chinese non-anaemic pregnant women, higher iron stores within the physiological range were associated with a higher risk of developing gestational diabetes [95]. Whilst some of the results from observational studies are mixed, broadly the data suggest that higher iron stores are associated with insulin resistance and T2DM. To date most of these studies have been carried out in Europe and the USA using predominantly Caucasian populations, and further work is needed to clarify whether the same patterns are seen in other ethnic groups.

A Mendelian randomisation study used three single nucleotide polymorphisms (SNPs) which are associated with iron status and investigated phenotypic associations. Although T2DM was not associated with any of the SNPs, hypercholesterolaemia was, suggesting a causal effect of iron status on lipid metabolism [96].

Prospective studies have gone a step further and shown a temporal association between iron status and insulin resistance, suggesting a causal relationship. In a meta-analysis of twelve prospective studies, ferritin and dietary haem intake were both associated with an increased risk of developing T2DM [97]. In another study of 509 individuals with a 7 year follow up period, ferritin and transferrin were prospectively positively associated with insulin resistance (as estimated by HOMA2-IR), adipocyte and hepatic insulin resistance and development of T2DM [98]. The EPIC-InterAct study contained 12,403 incident T2DM cases and 16,143 controls. Levels of ferritin and transferrin were prospectively associated with the development of T2DM [99]. Levels of ferritin were more predictive of T2DM occurrence in lean individuals. Counterintuitively, individuals with a high transferrin saturation were less likely to develop T2DM, suggesting that the relationship between iron stores and risk of diabetes is complex. One possible explanation is that people who are less able to transport iron bound to transferrin experience more tissue toxicity from free iron.

Ferritin rises in response to inflammation, and whilst studies adjusted for this, the measures used were crude and it is difficult to fully account for low-level systemic inflammation. However, the associations with other iron indices do suggest a true association between iron storage and obesity and T2DM.

Animal studies agree with the overall concept that iron is involved in the pathophysiology of metabolic disease. Mice fed an iron-rich diet developed increased insulin resistance leading to a 40% increase in fasting glucose, as well as three-fold higher triglycerides [5]. Conversely, obese (but not healthy weight) mice fed a low iron diet or treated with iron chelation had lower triglycerides and were protected against β-cell failure and insulin resistance [38].

It seems counterintuitive that obesity is associated with a risk for iron deficiency and is obviously a risk factor for metabolic disease, while iron excess appears to be associated with metabolic risk. The distribution of iron may partly explain this paradox; AT and liver are sensitive to iron deposition and serum iron deficiency does not necessarily reflect tissue iron status. Metabolic derangement seems often to be associated with a redistribution in iron stores characterised by a decrease in liver iron and an increase in iron stored in AT tissue [8]. Further research into this area is warranted, and studies using magnetic resonance imaging (MRI) techniques currently used to estimate cardiac and hepatic iron content [100] would be informative. New techniques in imaging analysis are being developed, such as the use of deep learning models to enhance characterisation of fat and iron content of multiple organs on MRI [101].

## Iron depletion and metabolic consequences of obesity in humans

Beyond disorders of pathological iron loading, the clinical evidence regarding iron depletion as a strategy to reduce metabolic risk is mixed, and overall there is insufficient evidence to support iron depletion as a therapeutic strategy.

NAFLD describes excess fat deposition in hepatocytes in the absence of non-metabolic causes of liver disease. Hepatic iron content is a predictor of progression to cirrhosis and hepatocellular carcinoma [102]. Therapeutic phlebotomy to cause iron depletion counteracts insulin resistance and improves lipid profiles in patients with hyperferritinaemia and NAFLD [6, 103].

In humans, metabolomics has suggested common pathways that differed between individuals with metabolic syndrome with and without hyperferritinaemia, which were also regulated by therapeutic phlebotomy [104]. In a randomised controlled trial, participants with metabolic syndrome who underwent phlebotomy had improvements in blood pressure, HbA1c and low-density lipoprotein (LDL)/high-density lipoprotein (LDL) ratio, but not in HOMA-IR [105].

If iron stores within the physiological range are associated with the pathophysiology of T2DM, one might expect the risk of T2DM to drop in those who regularly donate blood. A large study [106] which included more than 33,000 men who had given blood and which detected 1,168 new cases of T2DM over a 12 year follow up period found no protective effect of blood donation. However, men were classed as blood donors if they had given blood in the last 30 years at the time of recruitment, and despite the size of the study only a small number of participants were regular donors. Ferritin levels were only validated in a small subpopulation of donors. Nevertheless, these findings are echoed by a systematic review and meta-analysis of patients with DIOS and non-alcoholic fatty liver disease, which found no effect of iron depletion on HOMA-IR, insulin level, alanine aminotransferase (ALT), aspartate aminotransferase (AST) or liver fibrosis, even in those with high ferritin [107]. Similarly, treatment with an iron chelator does not appear to improve glycaemia in T2DM [108].

## Glucose and insulin modulate iron-regulatory pathways

As well as evidence that iron loading and aspects of the iron-regulatory pathway influence glucose and lipid metabolism, there is also data to suggest that glucose and insulin directly affect iron metabolism. Humans experience rapid changes in iron homoeostasis in response to an oral glucose ingestion; hepcidin levels increase and serum iron levels fall within 180 min [7]. Hyperglycaemia has been shown to decrease hepcidin release from a mouse insulinoma cell line [109]. Metabolomics studies have shown that iron overload affects multiple metabolic pathways. In a mouse model, dietary iron overload altered several metabolic pathways in peripheral blood and liver related to glucose homoeostasis and the Krebs cycle, hypothesised to be a compensatory response against increased oxidative stress [110].

Insulin itself has been shown to cause induction of hepcidin in the pancreatic β-cell [7] and in hepatocytes [7, 111]. Conversely, streptozotocin-induced β-cell failure in rats caused a decrease in hepatic hepcidin release, accompanied by enhanced intestinal iron absorption and increased hepatic iron deposition [111], which may be a mechanism for the pattern of iron deposition often seen in diabetes. Insulin may play a role in the distribution of iron stores between tissues; insulin encourages iron uptake into adipocytes by causing the redistribution of transferrin receptors to the cell surface [112].

Beyond insulin, other medications given for diabetes can also influence iron metabolism. Dapagliflozin, a sodium-glucose cotransporter-2 (SGLT2) inhibitor, caused an increase in EPO and ERFE and a fall in hepcidin when given to obese patients with T2DM for 12 weeks [113]. Treatment of mice with metformin led to decreased hepcidin and increased serum iron and transferrin saturation [114], although an observational and a double-blind study in humans did not replicate this [115].

Immuno-electron microscopy has revealed that hepcidin is contained exclusively within the insulin-containing secretory granule in the β-cell and is regulated by iron concentration [3]. In haemochromatosis, iron deposition in the endocrine pancreas is confined to β-cells and correlates with reduced secretory granule density [116], increased β-cell oxidative stress and apoptosis [117]. Forced expression of hepcidin partially rescued cells from glucotoxicity-related impaired insulin synthesis [109]. It may be that the local release of hepcidin ensures adequate iron supply and protects the β-cell from iron overload and thus toxicity in the context of hyperglycaemia.

## Iron and insulin resistance

The primary sites of insulin resistance are liver, skeletal muscle and white AT. The effects of iron status on AT insulin resistance have been discussed above.

### Iron and insulin resistance in liver

Between 30% and 60% of postprandial glucose uptake is by the liver [118]. Hepatic iron load, as determined by cross-sectional imaging, explains part of the variation between liver fat and insulin sensitivity [119]. Interestingly, in mice, a high fat diet led to alterations in tissue iron distribution, with a decrease in liver iron and an increase in iron stored in AT tissue [8]. In patients with NAFLD, insulin sensitivity improves after dietary iron restriction or phlebotomy [120]. In vitro experiments on a hepatocyte cell line suggest that iron depletion improves insulin sensitivity by increasing insulin receptor binding and upregulating pathways associated with glucose uptake [120].

### Iron and insulin resistance in muscle

Muscle contains 10–15% of the body’s iron stores, predominantly in the form of haem within myoglobin. Muscle is also a major site for glucose utilisation. As with all tissues, skeletal muscle has an absolute requirement for iron for normal function. Mice with skeletal-muscle specific deletion of the transferrin receptor, and thus iron deficiency in muscle, had impaired skeletal muscle metabolism but also developed a severe and lethal systemic metabolic phenotype with disappearance of fat pads and development of hepatic steatosis [121]. However, within the physiological range there is some evidence that low-normal iron stores might be metabolically beneficial. Glucose enters the muscle via the Glucose transporter type (GLUT)4 glucose transporter. Dietary iron restriction of rats led to upregulation of GLUT4 in skeletal muscle, associated with decreased fasting blood glucose levels [122]. In vitro, treatment of cultured myotubes with iron chelators (mirroring iron restriction) increased glucose uptake and utilisation via upregulation of GLUT1, but without changes in GLUT4 [123]. Iron overload in muscle leads to an autophagy defect, leading to accumulation of dysfunctional autolysosomes and to insulin resistance [124]. Mammalian target of rapamycin (mTOR) regulates cellular iron flux by affecting transferrin receptor-1 stability [125]. In mice, iron overload has been shown to lead to mTOR suppression mediated autophagy defect in skeletal muscle, causing insulin resistance [124].

Exercise leads to metabolically beneficial adaptations in skeletal muscle. Acutely, exercise improves insulin sensitivity and upregulates fat oxidation and lipolysis, and chronically, regular exercise reduces the risk of metabolic diseases including T2DM and NAFLD [126]. Iron deficiency is common in athletes, in particular in pre-menopausal women [127]. Exercise, independent of diet, affects iron metabolism. Circulating soluble transferrin receptor concentration decreased in people with obesity who lost weight through following a programme combining dietary modifications with resistance training, but not in those who lost weight through diet alone [128].

Within skeletal muscle, transcription of ERFE is increased after an hour of exercise by people with obesity, with or without T2DM [64]. A meta-analysis of sixteen studies showed that hepcidin increased with both aerobic and resistance exercise [129]. In adults with obesity, a 12 week exercise intervention consisting of either high-intensity or moderate-intensity interval training led to a decrease in whole body iron stores (as indicated by decreased ferritin) and reduced skeletal muscle iron stores, with a non-significant reduction in hepatic iron [130].

Exercise rapidly induces IL-6 release from skeletal muscle [131] which in turn stimulates transcription and release of hepcidin [31]. The degree of IL-6 rise during exercise is to some degree predictive of the degree of hepcidin rise, but the predominant factor dictating the degree of hepcidin rise with exercise is baseline iron status [132]. Leptin stimulates hepcidin release [46] and leptin levels and sensitivity are modulated by exercise [133, 134], but in vivo correlations between hepcidin and leptin in humans undergoing exercise programmes are inconsistent [135].

## Diet and iron regulation

### Macronutrient composition and iron regulation

An obvious mechanism that could link metabolic health and iron homoeostasis is diet. Dietary composition and energy intake are major determinants of metabolic health. As we have seen, glucose and lipid homoeostasis can be affected by the iron content of the diet. The composition of the diet, beyond simply the intake and bioavailability of iron consumed, can also impact iron regulation. High dietary fat intake may change hepatic iron metabolism. Rats fed a high fat diet had increased hepatic activity of iron-regulatory protein 1 (IRP1), increased transferrin receptor-1 expression and downregulated ferritin and ferroportin, which preceded hepatic iron deposition [136].

At least in the context of exercise related hepcidin release, macronutrient intake does not appear to be as important for determining hepcidin response as overall energy intake [137]. Hepcidin is upregulated in response to a negative energy balance. Greater increases in hepcidin occur if exercise is combined with a low energy diet compared with adequate energy intake [137, 138].

AT iron status might affect dietary fat absorption. A recent study showed that, unexpectedly, mice with adipocyte-specific deletion of transferrin receptor-1 leading to local iron deficiency had impaired absorption of lipids from the intestine [40]. These findings were replicated in mice receiving transplanted iron deficient AT [40].

### Iron supplementation and the microbiome

Dietary iron can affect the microbiome, and conversely the microbiome can affect dietary iron absorption [139]. Iron is essential for the survival of the majority of bacteria colonising the human intestinal tract, although some are iron independent [140]. Several studies have demonstrated short term changes of the microbiome with iron supplementation with a general move towards a more pathogenic and inflammatory profile [141, 142]. Whilst the majority of iron absorption takes place in the duodenum, rodents lacking a microbiome exhibit alterations in iron-regulatory genes in the colon [143] and have a reduced capacity to absorb dietary iron [144]. The intestinal microbiome is recognised to be an important regulator of metabolic health [145], but the extent to which microbiome shifts due to oral iron impact on whole body glucose and lipid metabolism is not yet clear.

### Vitamin D and iron metabolism

Vitamin D deficiency is commonly associated with obesity [24] and may worsen insulin resistance [146]. Volunteers given a single dose of vitamin D downregulated hepcidin within 24 h [147]. Mechanistically, treatment of cultured hepatocytes with 25 or 1, 25 vitamin D caused upregulation of ferroportin and downregulation of hepcidin mRNA and protein by direct transcriptional suppression [147].

### Iron and gluconeogenesis

Hepatic gluconeogenesis acts to maintain glucose supply during starvation. In diabetes, hepatic gluconeogenesis is inappropriately active and contributes to hyperglycaemia [148]. In response to starvation in mice, hepcidin was upregulated directly by the gluconeogenic signals PPARGC1A and CREB3L3 (both glucagon responsive), leading to an increase in hepatic iron deposition [149]. Gluconeogenesis is entrained to circadian rhythm according to feeding patterns [150]. Iron intake also modulates gluconeogenesis; in mice, dietary iron intake was shown to affect the circadian rhythm of gluconeogenesis, such that a high iron diet suppressed gluconeogenesis via altering haem synthesis that then acted on Rev-Erbα, a haem-binding transcription factor and a key regulator of the circadian clock [151].

## Areas for future research

Much remains to be discovered in this fascinating area. Validation and use of non-invasive techniques for estimating iron content in AT and other tissues will allow us to explore the impact of iron distribution on lipid and glucose metabolism, and to explore how tissue iron distribution is related to other factors such as body fat distribution. Pharmacological agents targeting aspects of the iron pathway, such as hepcidin and ferroportin, have been developed and are being assessed as therapeutic agents for iron-related diseases. Future studies should assess the effect of these on glucose and lipid metabolism.

## Conclusions

In this review we have summarised the complex bidirectional relationship between iron metabolism on the one hand and body fat, glucose and lipid metabolism on the other. Alterations in iron status and metabolism, both pathologically and within the normal physiological range, affect fat deposition and distribution and metabolic risk. Equally, systemic glucose, lipid and insulin affect iron-regulatory pathways. It is possible that these pathways intertwined during evolution to support maintenance of adequate iron stores during periods of nutritional scarcity, and vice versa. A detailed understanding of mechanisms linking iron metabolism and metabolic risk may provide the foundation for future therapeutic interventions.

## References

[1] Andreini C, Putignano V, Rosato A, Banci L (2018). The human iron-proteome. Metallomics..

[2] Bekri S, Gual P, Anty R, Luciani N, Dahman M, Ramesh B (2006). Increased adipose tissue expression of hepcidin in severe obesity is independent from diabetes and NASH. Gastroenterology..

[3] Kulaksiz H, Fein E, Redecker P, Stremmel W, Adler G, Cetin Y (2008). Pancreatic beta-cells express hepcidin, an iron-uptake regulatory peptide. J Endocrinol.

[4] Oshaug A, Bugge KH, Bjonnes CH, Borchiohnsen B, Neslein IL (1995). Associations between Serum Ferritin and Cardiovascular Risk-Factors in Healthy-Young Men—a Cross-Sectional Study. Eur J Clin Nutr.

[5] Dongiovanni P, Ruscica M, Rametta R, Recalcati S, Steffani L, Gatti S (2013). Dietary iron overload induces visceral adipose tissue insulin resistance. Am J Pathol.

[6] Valenti L, Remondini E, Fracanzani AL, Spada A, Colombo S, Guzzo A (2005). Effect of iron depletion on liver function and insulin resistance in patients with NASH and fatty liver. Hepatology..

[7] Aigner E, Felder TK, Oberkofler H, Hahne P, Auer S, Soyal S (2013). Glucose acts as a regulator of serum iron by increasing serum hepcidin concentrations. J Nutr Biochem.

[8] Varghese J, James JV, Anand R, Narayanasamy M, Rebekah G, Ramakrishna B (2020). Development of insulin resistance preceded major changes in iron homeostasis in mice fed a high-fat diet. J Nutr Biochem.

[9] Abbas MA, Abraham D, Kushner JP, McClain DA (2014). Anti-obesity and pro-diabetic effects of hemochromatosis. Obesity.

[10] Oexle H, Gnaiger E, Weiss G (1999). Iron-dependent changes in cellular energy metabolism: influence on citric acid cycle and oxidative phosphorylation. Biochim Biophys Acta.

[11] Volani C, Doerrier C, Demetz E, Haschka D, Paglia G, Lavdas AA (2017). Dietary iron loading negatively affects liver mitochondrial function. Metallomics..

[12] Coffey R, Ganz T (2017). Iron homeostasis: An anthropocentric perspective. J Biol Chem.

[13] Andriopoulos B, Corradini E, Xia Y, Faasse SA, Chen S, Grgurevic L (2009). BMP6 is a key endogenous regulator of hepcidin expression and iron metabolism. Nat Genet.

[14] Silvestri L, Nai A, Dulja A, Pagani A (2019). Hepcidin and the BMP-SMAD pathway: An unexpected liaison. Vitam Horm.

[15] Nemeth E, Rivera S, Gabayan V, Keller C, Taudorf S, Pedersen BK (2004). IL-6 mediates hypoferremia of inflammation by inducing the synthesis of the iron regulatory hormone hepcidin. J Clin Investig.

[16] Ganz T, Nemeth E (2015). Iron homeostasis in host defence and inflammation. Nat Rev Immunol.

[17] Arezes J, Foy N, McHugh K, Sawant A, Quinkert D, Terraube V (2018). Erythroferrone inhibits the induction of hepcidin by BMP6. Blood..

[18] Kautz L, Jung G, Valore EV, Rivella S, Nemeth E, Ganz T (2014). Identification of erythroferrone as an erythroid regulator of iron metabolism. Nat Genet.

[19] Wenzel BJ, Stults HB, Mayer J (1962). Hypoferraemia in obese adolescents. Lancet..

[20] Nead KG, Halterman JS, Kaczorowski JM, Auinger P, Weitzman M (2004). Overweight children and adolescents: a risk group for iron deficiency. Pediatrics..

[21] Cercamondi CI, Stoffel NU, Moretti D, Zoller T, Swinkels DW, Zeder C (2021). Iron homeostasis during anemia of inflammation: a prospective study of patients with tuberculosis. Blood..

[22] Tussing-Humphreys LM, Nemeth E, Fantuzzi G, Freels S, Holterman AX, Galvani C (2010). Decreased serum hepcidin and improved functional iron status 6 months after restrictive bariatric surgery. Obesity..

[23] Vaquero MP, Martinez-Suarez M, Garcia-Quismondo A, del Canizo FJ, Sanchez-Muniz FJ (2021). Diabesity negatively affects transferrin saturation and iron status. The DICARIVA study. Diabetes Res Clin Pract.

[24] Lefebvre P, Letois F, Sultan A, Nocca D, Mura T, Galtier F (2014). Nutrient deficiencies in patients with obesity considering bariatric surgery: a cross-sectional study. Surg Obes Relat Dis.

[25] Wells JC, Sawaya AL, Wibaek R, Mwangome M, Poullas MS, Yajnik CS (2020). The double burden of malnutrition: aetiological pathways and consequences for health. Lancet..

[26] Chambers EC, Heshka S, Gallagher D, Wang J, Pi-Slinyer FX, Pierson RN (2006). Serum iron and body fat distribution in a multiethnic cohort of adults living in New York City. J Am Diet Assoc.

[27] Gillum RF (2001). Association of serum ferritin and indices of body fat distribution and obesity in Mexican American men - the Third National Health and Nutrition Examination Survey. Int J Obesity.

[28] Iwasaki T, Nakajima A, Yoneda M, Yamada Y, Mukasa K, Fujita K (2005). Serum ferritin is associated with visceral fat area and subcutaneous fat area. Diabetes Care.

[29] Stoffel NU, El-Mallah C, Herter-Aeberli I, Bissani N, Wehbe N, Obeid O (2020). The effect of central obesity on inflammation, hepcidin, and iron metabolism in young women. Int J Obes.

[30] Olefsky JM, Glass CK (2010). Macrophages, inflammation, and insulin resistance. Annu Rev Physiol.

[31] Wrighting DM, Andrews NC (2006). Interleukin-6 induces hepcidin expression through STAT3. Blood..

[32] Yang Q, Jian J, Katz S, Abramson SB, Huang X (2012). 17beta-Estradiol inhibits iron hormone hepcidin through an estrogen responsive element half-site. Endocrinology..

[33] Kawai T, Autieri MV, Scalia R (2021). Adipose tissue inflammation and metabolic dysfunction in obesity. Am J Physiol Cell Physiol.

[34] Hotamisligil GS (2006). Inflammation and metabolic disorders. Nature..

[35] Cepeda-Lopez AC, Allende-Labastida J, Melse-Boonstra A, Osendarp SJ, Herter-Aeberli I, Moretti D (2016). The effects of fat loss after bariatric surgery on inflammation, serum hepcidin, and iron absorption: a prospective 6-mo iron stable isotope study. Am J Clin Nutr.

[36] Moreno-Navarrete JM, Novelle MG, Catalan V, Ortega F, Moreno M, Gomez-Ambrosi J (2014). Insulin resistance modulates iron-related proteins in adipose tissue. Diabetes Care.

[37] Tajima S, Ikeda Y, Sawada K, Yamano N, Horinouchi Y, Kihira Y (2012). Iron reduction by deferoxamine leads to amelioration of adiposity via the regulation of oxidative stress and inflammation in obese and type 2 diabetes KKAy mice. Am J Physiol Endocrinol Metab.

[38] Cooksey RC, Jones D, Gabrielsen S, Huang J, Simcox JA, Luo B (2010). Dietary iron restriction or iron chelation protects from diabetes and loss of beta-cell function in the obese (ob/ob lep-/-) mouse. Am J Physiol Endocrinol Metab.

[39] Tang Y, Wang D, Zhang H, Zhang Y, Wang J, Qi R (2021). Rapid responses of adipocytes to iron overload increase serum TG level by decreasing adiponectin. J Cell Physiol.

[40] Zhang Z, Funcke JB, Zi Z, Zhao S, Straub LG, Zhu Y (2021). Adipocyte iron levels impinge on a fat-gut crosstalk to regulate intestinal lipid absorption and mediate protection from obesity. Cell Metab.

[41] Sinha S, Pereira-Reis J, Guerra A, Rivella S, Duarte D (2021). The Role of Iron in Benign and Malignant Hematopoiesis. Antioxid Redox Signal.

[42] Rumberger JM, Peters T, Burrington C, Green A (2004). Transferrin and iron contribute to the lipolytic effect of serum in isolated adipocytes. Diabetes..

[43] Gabrielsen JS, Gao Y, Simcox JA, Huang J, Thorup D, Jones D (2012). Adipocyte iron regulates adiponectin and insulin sensitivity. J Clin Investig.

[44] Gao Y, Li Z, Gabrielsen JS, Simcox JA, Lee SH, Jones D (2015). Adipocyte iron regulates leptin and food intake. J Clin Investig.

[45] Katsiki N, Mantzoros C, Mikhailidis DP (2017). Adiponectin, lipids and atherosclerosis. Curr Opin Lipidol.

[46] Chung B, Matak P, McKie AT, Sharp P (2007). Leptin increases the expression of the iron regulatory hormone hepcidin in HuH7 human hepatoma cells. J Nutr.

[47] Orr JS, Kennedy A, Anderson-Baucum EK, Webb CD, Fordahl SC, Erikson KM (2014). Obesity alters adipose tissue macrophage iron content and tissue iron distribution. Diabetes..

[48] Hubler MJ, Erikson KM, Kennedy AJ, Hasty AH (2018). MFe(hi) adipose tissue macrophages compensate for tissue iron perturbations in mice. Am J Physiol Cell Physiol.

[49] McBride HM, Neuspiel M, Wasiak S (2006). Mitochondria: More than just a powerhouse. Curr Biol.

[50] Mills EL, Kelly B, O’Neill LAJ (2017). Mitochondria are the powerhouses of immunity. Nat Immunol.

[51] Walter PB, Knutson MD, Paler-Martinez A, Lee S, Xu Y, Viteri FE (2002). Iron deficiency and iron excess damage mitochondria and mitochondrial DNA in rats. Proc Natl Acad Sci USA.

[52] Heinonen S, Jokinen R, Rissanen A, Pietilainen KH (2020). White adipose tissue mitochondrial metabolism in health and in obesity. Obes Rev.

[53] Kusminski CM, Holland WL, Sun K, Park J, Spurgin SB, Lin Y (2012). MitoNEET-driven alterations in adipocyte mitochondrial activity reveal a crucial adaptive process that preserves insulin sensitivity in obesity. Nat Med.

[54] Cannon B, Nedergaard J (2004). Brown adipose tissue: function and physiological significance. Physiol Rev.

[55] Cousin B, Cinti S, Morroni M, Raimbault S, Ricquier D, Penicaud L (1992). Occurrence of brown adipocytes in rat white adipose tissue: molecular and morphological characterization. J Cell Sci.

[56] Cypess AM, Lehman S, Williams G, Tal I, Rodman D, Goldfine AB (2009). Identification and importance of brown adipose tissue in adult humans. N Engl J Med.

[57] Rosenzweig PH, Volpe SL (1999). Iron, thermoregulation, and metabolic rate. Crit Rev Food Sci Nutr.

[58] Blasco G, Puig J, Daunis IEJ, Molina X, Xifra G, Fernandez-Aranda F (2014). Brain iron overload, insulin resistance, and cognitive performance in obese subjects: a preliminary MRI case-control study. Diabetes Care.

[59] Lawless JW, Latham MC, Stephenson LS, Kinoti SN, Pertet AM (1994). Iron supplementation improves appetite and growth in anemic Kenyan primary school children. J Nutr.

[60] Soliman AT, De Sanctis V, Yassin M, Wagdy M, Soliman N (2017). Chronic anemia and thyroid function. Acta Biomed.

[61] Soliman AT, De Sanctis V, Yassin M, Adel A (2017). Growth and Growth hormone - Insulin Like Growth Factor -I (GH-IGF-I) Axis in Chronic Anemias. Acta Biomed.

[62] Seldin MM, Peterson JM, Byerly MS, Wei Z, Wong GW (2012). Myonectin (CTRP15), a novel myokine that links skeletal muscle to systemic lipid homeostasis. J Biol Chem.

[63] Pourranjbar M, Arabnejad N, Naderipour K, Rafie F (2018). Effects of Aerobic Exercises on Serum Levels of Myonectin and Insulin Resistance in Obese and Overweight Women. J Med Life.

[64] Sabaratnam R, Wojtaszewski JFP, Hojlund K (2022). Factors mediating exercise-induced organ crosstalk. Acta Physiol.

[65] Li L, Wang QQ, Qin CK (2020). Serum myonectin is increased after laparoscopic sleeve gastrectomy. Ann Clin Biochem.

[66] Li K, Liao X, Wang K, Mi Q, Zhang T, Jia Y (2018). Myonectin Predicts the Development of Type 2 Diabetes. J Clin Endocrinol Metab.

[67] Denton NF, Eghleilib M, Al-Sharifi S, Todorcevic M, Neville MJ, Loh N (2019). Bone morphogenetic protein 2 is a depot-specific regulator of human adipogenesis. Int J Obes.

[68] Katz O, Stuible M, Golishevski N, Lifshitz L, Tremblay ML, Gassmann M (2010). Erythropoietin treatment leads to reduced blood glucose levels and body mass: insights from murine models. J Endocrinol.

[69] Li J, Yang M, Yu Z, Tian J, Du S, Ding H (2019). Kidney-secreted erythropoietin lowers lipidemia via activating JAK2-STAT5 signaling in adipose tissue. EBioMedicine..

[70] Foskett A, Alnaeeli M, Wang L, Teng R, Noguchi CT (2011). The effects of erythropoietin dose titration during high-fat diet-induced obesity. J Biomed Biotechnol.

[71] Vinberg M, Hojman P, Pedersen BK, Kessing LV, Miskowiak KW (2018). Effects of erythropoietin on body composition and fat-glucose metabolism in patients with affective disorders. Acta Neuropsychiatr.

[72] Teng R, Gavrilova O, Suzuki N, Chanturiya T, Schimel D, Hugendubler L (2011). Disrupted erythropoietin signalling promotes obesity and alters hypothalamus proopiomelanocortin production. Nat Commun.

[73] Liu Y, Luo B, Shi R, Wang J, Liu Z, Liu W (2015). Nonerythropoietic Erythropoietin-Derived Peptide Suppresses Adipogenesis, Inflammation, Obesity and Insulin Resistance. Sci Rep.

[74] Root HF (1929). Insulin resistance and bronze diabetes. N Engl J Med.

[75] Fibach E, Rachmilewitz EA (2017). Pathophysiology and treatment of patients with beta-thalassemia—an update. F1000Res.

[76] Bahar A, Shekarriz R, Janbabai G, Shirzad R, Aarabi M, Kashi Z (2015). Insulin resistance, impaired glucose tolerance and alpha-thalassemia carrier state. J Diabetes Metab Disord.

[77] Bahar A, Kashi Z, Sohrab M, Kosaryan M, Janbabai G (2012). Relationship between beta-globin gene carrier state and insulin resistance. J Diabetes Metab Disord.

[78] Chatterjee R, Bajoria R (2009). New concept in natural history and management of diabetes mellitus in thalassemia major. Hemoglobin.

[79] Le Gac G, Ferec C (2005). The molecular genetics of haemochromatosis. Eur J Hum Genet.

[80] Pilling LC, Tamosauskaite J, Jones G, Wood AR, Jones L, Kuo CL (2019). Common conditions associated with hereditary haemochromatosis genetic variants: cohort study in UK Biobank. BMJ..

[81] McClain DA, Abraham D, Rogers J, Brady R, Gault P, Ajioka R (2006). High prevalence of abnormal glucose homeostasis secondary to decreased insulin secretion in individuals with hereditary haemochromatosis. Diabetologia..

[82] Solanas-Barca M, Mateo-Gallego R, Calmarza P, Jarauta E, Bea AM, Cenarro A (2009). Mutations in HFE causing hemochromatosis are associated with primary hypertriglyceridemia. J Clin Endocrinol Metab.

[83] Casanova-Esteban P, Guiral N, Andres E, Gonzalvo C, Mateo-Gallego R, Giraldo P (2011). Effect of phlebotomy on lipid metabolism in subjects with hereditary hemochromatosis. Metabolism Clin Exp.

[84] Mateo-Gallego R, Lacalle L, Perez-Calahorra S, Marco-Benedi V, Recasens V, Padron N (2018). Efficacy of repeated phlebotomies in hypertriglyceridemia and iron overload: A prospective, randomized, controlled trial. J Clin Lipidol.

[85] Mayr R, Janecke AR, Schranz M, Griffiths WJ, Vogel W, Pietrangelo A (2010). Ferroportin disease: a systematic meta-analysis of clinical and molecular findings. J Hepatol.

[86] Marques VB, Leal MAS, Mageski JGA, Fidelis HG, Nogueira BV, Vasquez EC (2019). Chronic iron overload intensifies atherosclerosis in apolipoprotein E deficient mice: Role of oxidative stress and endothelial dysfunction. Life Sci.

[87] Lieb M, Palm U, Hock B, Schwarz M, Domke I, Soyka M (2011). Effects of alcohol consumption on iron metabolism. Am J Drug Alcohol Abuse.

[88] Chapman RW, Morgan MY, Laulicht M, Hoffbrand AV, Sherlock S (1982). Hepatic iron stores and markers of iron overload in alcoholics and patients with idiopathic hemochromatosis. Dig Dis Sci.

[89] Gerjevic LN, Liu N, Lu S, Harrison-Findik DD (2012). Alcohol Activates TGF-Beta but Inhibits BMP Receptor-Mediated Smad Signaling and Smad4 Binding to Hepcidin Promoter in the Liver. Int J Hepatol.

[90] Harrison-Findik DD, Schafer D, Klein E, Timchenko NA, Kulaksiz H, Clemens D (2006). Alcohol metabolism-mediated oxidative stress down-regulates hepcidin transcription and leads to increased duodenal iron transporter expression. J Biol Chem.

[91] Bonfils L, Ellervik C, Friedrich N, Linneberg A, Sandholt CH, Jorgensen ME (2015). Fasting serum levels of ferritin are associated with impaired pancreatic beta cell function and decreased insulin sensitivity: a population-based study. Diabetologia..

[92] Ndevahoma F, Mukesi M, Dludla PV, Nkambule BB, Nepolo EP, Nyambuya TM (2021). Body weight and its influence on hepcidin levels in patients with type 2 diabetes: A systematic review and meta-analysis of clinical studies. Heliyon..

[93] Vela D, Leshoski J, Vela Z, Jakupaj M, Mladenov M, Sopi RB (2017). Insulin treatment corrects hepcidin but not YKL-40 levels in persons with type 2 diabetes mellitus matched by body mass index, waist-to-height ratio, C-reactive protein and Creatinine. BMC Endocr Disord.

[94] Sam AH, Busbridge M, Amin A, Webber L, White D, Franks S (2013). Hepcidin levels in diabetes mellitus and polycystic ovary syndrome. Diabetic Med J Br Diabetic Assoc.

[95] Lao TT, Chan PL, Tam KF (2001). Gestational diabetes mellitus in the last trimester—a feature of maternal iron excess?. Diabetic Med J Br Diabetic Assoc.

[96] Gill D, Benyamin B, Moore LSP, Monori G, Zhou A, Koskeridis F (2019). Associations of genetically determined iron status across the phenome: A mendelian randomization study. PLoS Med.

[97] Kunutsor SK, Apekey TA, Walley J, Kain K (2013). Ferritin levels and risk of type 2 diabetes mellitus: an updated systematic review and meta-analysis of prospective evidence. Diabetes Metab Res Rev.

[98] Wlazlo N, van Greevenbroek MMJ, Ferreira I, Jansen EHJM, Feskens EJM, van der Kallen CJH (2015). Iron metabolism is prospectively associated with insulin resistance and glucose intolerance over a 7-year follow-up period: the CODAM study. Acta Diabetol.

[99] Podmore C, Meidtner K, Schulze MB, Scott RA, Ramond A, Butterworth AS (2016). Association of Multiple Biomarkers of Iron Metabolism and Type 2 Diabetes: The EPIC-InterAct Study. Diabetes Care.

[100] Wood JC (2011). Impact of iron assessment by MRI. Hematol Am Soc Hematol Educ Prog.

[101] Liu Y, Basty N, Whitcher B, Bell JD, Sorokin EP, van Bruggen N (2021). Genetic architecture of 11 organ traits derived from abdominal MRI using deep learning. Elife.

[102] Corradini E, Pietrangelo A (2012). Iron and steatohepatitis. J Gastroenterol Hepatol.

[103] Jaruvongvanich V, Riangwiwat T, Sanguankeo A, Upala S (2016). Outcome of phlebotomy for treating nonalcoholic fatty liver disease: A systematic review and meta-analysis. Saudi J Gastroenterol.

[104] Stechemesser L, Eder SK, Wagner A, Patsch W, Feldman A, Strasser M (2017). Metabolomic profiling identifies potential pathways involved in the interaction of iron homeostasis with glucose metabolism. Mol Metab.

[105] Houschyar KS, Ludtke R, Dobos GJ, Kalus U, Broecker-Preuss M, Rampp T (2012). Effects of phlebotomy-induced reduction of body iron stores on metabolic syndrome: results from a randomized clinical trial. BMC Med.

[106] Jiang R, Ma J, Ascherio A, Stampfer MJ, Willett WC, Hu FB (2004). Dietary iron intake and blood donations in relation to risk of type 2 diabetes in men: a prospective cohort study. Am J Clin Nutr.

[107] Murali AR, Gupta A, Brown K (2018). Systematic review and meta-analysis to determine the impact of iron depletion in dysmetabolic iron overload syndrome and non-alcoholic fatty liver disease. Hepatol Res.

[108] Redmon JB, Pyzdrowski KL, Robertson RP (1993). No Effect of Deferoxamine Therapy on Glucose-Homeostasis and Insulin-Secretion in Individuals with Niddm and Elevated Serum Ferritin. Diabetes..

[109] Mao X, Chen H, Tang J, Wang L, Shu T (2017). Hepcidin links gluco-toxicity to pancreatic beta cell dysfunction by inhibiting Pdx-1 expression. Endocr Connect.

[110] Volani C, Paglia G, Smarason SV, Pramstaller PP, Demetz E, Pfeifhofer-Obermair C (2018). Metabolic Signature of Dietary Iron Overload in a Mouse Model. Cells..

[111] Wang HY, Li HX, Jiang X, Shi WC, Shen ZL, Li M (2014). Hepcidin Is Directly Regulated by Insulin and Plays an Important Role in Iron Overload in Streptozotocin- Induced Diabetic Rats. Diabetes..

[112] Davis RJ, Corvera S, Czech MP (1986). Insulin stimulates cellular iron uptake and causes the redistribution of intracellular transferrin receptors to the plasma membrane. J Biol Chem.

[113] Ghanim H, Abuaysheh S, Hejna J, Green K, Batra M, Makdissi A (2020). Dapagliflozin Suppresses Hepcidin And Increases Erythropoiesis. J Clin Endocr Metab.

[114] Wang MJ, Xin H, Tang WB, Li YM, Zhang ZY, Fan LL (2017). AMPK Serves as a Therapeutic Target Against Anemia of Inflammation. Antioxid Redox. Sign..

[115] Suarez-Ortegon MF, Moreno M, Arbelaez A, Xifra G, Mosquera M, Moreno-Navarrete JM (2015). Circulating hepcidin in type 2 diabetes: A multivariate analysis and double blind evaluation of metformin effects. Mol Nutr Food Res.

[116] Rahier J, Loozen S, Goebbels RM, Abrahem M (1987). The haemochromatotic human pancreas: a quantitative immunohistochemical and ultrastructural study. Diabetologia..

[117] Cooksey RC, Jouihan HA, Ajioka RS, Hazel MW, Jones DL, Kushner JP (2004). Oxidative stress, beta-cell apoptosis, and decreased insulin secretory capacity in mouse models of hemochromatosis. Endocrinology..

[118] Adeva-Andany MM, Perez-Felpete N, Fernandez-Fernandez C, Donapetry-Garcia C, Pazos-Garcia C (2016). Liver glucose metabolism in humans. Bioscience Rep.

[119] Haap M, Machann J, von Friedeburg C, Schick F, Stefan N, Schwenzer NF (2011). Insulin Sensitivity and Liver Fat: Role of Iron Load. J Clin Endocr Metab.

[120] Fargion S, Dongiovanni P, Guzzo A, Colombo S, Valenti L, Fracanzani AL (2005). Iron and insulin resistance. Aliment Pharm Therap.

[121] Barrientos T, Laothamatas I, Koves TR, Soderblom EJ, Bryan M, Moseley MA (2015). Metabolic Catastrophe in Mice Lacking Transferrin Receptor in Muscle. EBioMedicine..

[122] Mehdad A, Campos NA, Arruda SF, Siqueira EMD (2015). Iron deprivation may enhance insulin receptor and Glut4 transcription in skeletal muscle of adult rats. J Nutr Health Aging.

[123] Potashnik R, Kozlovsky N, Ben-Ezra S, Rudich A, Bashan N (1995). Regulation of glucose transport and GLUT-1 expression by iron chelators in muscle cells in culture. Am J Physiol.

[124] Jahng JWS, Alsaadi RM, Palanivel R, Song E, Hipolito VEB, Sung HK (2019). Iron overload inhibits late stage autophagic flux leading to insulin resistance. EMBO Rep.

[125] Bayeva M, Khechaduri A, Puig S, Chang HC, Patial S, Blackshear PJ (2012). mTOR regulates cellular iron homeostasis through tristetraprolin. Cell Metab.

[126] Thyfault JP, Bergouignan A (2020). Exercise and metabolic health: beyond skeletal muscle. Diabetologia..

[127] McKay AKA, Pyne DB, Burke LM, Peeling P (2020). Iron Metabolism: Interactions with Energy and Carbohydrate Availability. Nutrients..

[128] Fernandez-Real JM, Izquierdo M, Moreno-Navarrete JM, Gorostiaga E, Ortega F, Martinez C (2009). Circulating soluble transferrin receptor concentration decreases after exercise-induced improvement of insulin sensitivity in obese individuals. Int J Obes.

[129] Larsuphrom P, Latunde-Dada GO (2021). Association of Serum Hepcidin Levels with Aerobic and Resistance Exercise: A Systematic Review. Nutrients..

[130] Ryan BJ, Foug KL, Gioscia-Ryan RA, Varshney P, Ludzki AC, Ahn C (2021). Exercise training decreases whole-body and tissue iron storage in adults with obesity. Exp Physiol.

[131] Pedersen BK, Steensberg A, Schjerling P (2001). Exercise and interleukin-6. Curr Opin Hematol.

[132] Peeling P, McKay AKA, Pyne DB, Guelfi KJ, McCormick RH, Laarakkers CM (2017). Factors influencing the post-exercise hepcidin-25 response in elite athletes. Eur J Appl Physiol.

[133] Tremblay A, Dutheil F, Drapeau V, Metz L, Lesour B, Chapier R (2019). Long-term effects of high-intensity resistance and endurance exercise on plasma leptin and ghrelin in overweight individuals: the RESOLVE Study. Appl Physiol Nutr Metab.

[134] Bobbert T, Mai K, Brechtel L, Schulte HM, Weger B, Pfeiffer AF (2012). Leptin and endocrine parameters in marathon runners. Int J Sports Med.

[135] Nirengi S, Taniguchi H, Ishibashi A, Fujibayashi M, Akiyama N, Kotani K (2021). Comparisons Between Serum Levels of Hepcidin and Leptin in Male College-Level Endurance Runners and Sprinters. Front Nutr.

[136] Meli R, Mattace Raso G, Irace C, Simeoli R, Di Pascale A, Paciello O (2013). High Fat Diet Induces Liver Steatosis and Early Dysregulation of Iron Metabolism in Rats. PLoS ONE.

[137] Pasiakos SM, Margolis LM, Murphy NE, McClung HL, Martini S, Gundersen Y (2016). Effects of exercise mode, energy, and macronutrient interventions on inflammation during military training. Physiol Rep.

[138] Ishibashi A, Kojima C, Tanabe Y, Iwayama K, Hiroyama T, Tsuji T (2020). Effect of low energy availability during three consecutive days of endurance training on iron metabolism in male long distance runners. Physiol Rep.

[139] Yilmaz B, Li H (2018). Gut Microbiota and Iron: The Crucial Actors in Health and Disease. Pharmaceuticals.

[140] Archibald F (1983). Lactobacillus-Plantarum, an Organism Not Requiring Iron. Fems Microbiol Lett.

[141] Jaeggi T, Kortman GA, Moretti D, Chassard C, Holding P, Dostal A (2015). Iron fortification adversely affects the gut microbiome, increases pathogen abundance and induces intestinal inflammation in Kenyan infants. Gut..

[142] Zimmermann MB, Chassard C, Rohner F, N’Goran EK, Nindjin C, Dostal A (2010). The effects of iron fortification on the gut microbiota in African children: a randomized controlled trial in Cote d’Ivoire. Am J Clin Nutr.

[143] Deschemin JC, Noordine ML, Remot A, Willemetz A, Afif C, Canonne-Hergaux F (2016). The microbiota shifts the iron sensing of intestinal cells. FASEB J.

[144] Reddy BS, Pleasants JR, Wostmann BS (1972). Effect of intestinal microflora on iron and zinc metabolism, and on activities of metalloenzymes in rats. J Nutr.

[145] Ridaura VK, Faith JJ, Rey FE, Cheng J, Duncan AE, Kau AL (2013). Gut microbiota from twins discordant for obesity modulate metabolism in mice. Science..

[146] Talaei A, Mohamadi M, Adgi Z (2013). The effect of vitamin D on insulin resistance in patients with type 2 diabetes. Diabetol Metab Syndr.

[147] Bacchetta J, Zaritsky JJ, Sea JL, Chun RF, Lisse TS, Zavala K (2014). Suppression of iron-regulatory hepcidin by vitamin D. J Am Soc Nephrol.

[148] Zhang X, Yang S, Chen J, Su Z (2018). Unraveling the Regulation of Hepatic Gluconeogenesis. Front Endocrinol.

[149] Vecchi C, Montosi G, Garuti C, Corradini E, Sabelli M, Canali S (2014). Gluconeogenic signals regulate iron homeostasis via hepcidin in mice. Gastroenterology..

[150] Stokkan KA, Yamazaki S, Tei H, Sakaki Y, Menaker M (2001). Entrainment of the circadian clock in the liver by feeding. Science..

[151] Simcox JA, Mitchell TC, Gao Y, Just SF, Cooksey R, Cox J (2015). Dietary iron controls circadian hepatic glucose metabolism through heme synthesis. Diabetes..

